# Hæmorrhoids and Prolapsus of the Rectum; Their Pathology and Treatment

**Published:** 1860-07

**Authors:** 


ART. VIII.—*Hæmorrhoids and Prolapsus of the Rectum; their Pathology and Treatment; with special reference to the Application of Nitric Acid.* By HENRY SMITH, F.R.C.S., etc., etc. Second edition. 18mo., pp. 108. London, 1860.

MR. SMITH, the author of this little volume, is evidently a young man, working for practice and position. The work, however, is really a very good one, perfectly scientific, well written, and replete with sound practical sense. Founded essentially upon essays originally published in the *London Times and Gazette*, it was at first intended merely to show the value of the local application of nitric acid in certain forms of piles and prolapse of the rectum, a mode of treatment which, Mr. Smith thinks, is still not sufficiently appreciated by the majority of surgeons. The present edition has been considerably enlarged by the addition of new text and new cases, and may therefore be regarded as a tolerably satisfactory monograph upon these two affections.

A cursory examination of the pathological portions of Mr. Smith's little book has not revealed to us any new light in regard to the etiology and morbid anatomy of hæmorrhoids and rectal prolapse, nor have we discovered any novelties in the treatment of these two diseases. The nitric acid treatment for piles, originally proclaimed by the late Mr. Houston, of Dublin, and so much lauded by several recent writers, is evidently a great favorite with the author, although it is but just to say that he does not award to it exclusive preference. On the contrary, he distinctly declares that it is applicable only to the milder varieties of the affection, leaving the more ancient and severe cases to be dealt with by the ligature. Excision he, of course, in common with all other respectable surgeons, pointedly condemns.

In using the nitric acid, none but the strongest and purest article should be trusted; it should be applied by means of a piece of soft wood, cut flat at the end, the bowel being previously forced down by straining or by an enema, and the surface carefully wiped. The touched part, as well as the adjacent border, is then thoroughly oiled, and the prolapsed bowel pressed up into its natural position. Several applications may be required before a final cure is effected. An anodyne is administered after the operation, to prevent peristaltic action, and the case is treated upon strictly antiphlogistic principles.

We hope that we shall have the pleasure to meet Mr. Smith soon again in other works upon less trite subjects, and of a more stately form. We do not think that the interests of science, or of humanity either, would have materially suffered if the present little book had never seen the light. We are sure that there are five hundred works before the profession from which the student could glean all that, if indeed not a good deal more than, is to be found in this volume.

